# The impact of early phase price agreements on prices of orphan drugs

**DOI:** 10.1186/s12913-021-06208-7

**Published:** 2021-03-12

**Authors:** Mark Nuijten, Philippe Van Wilder

**Affiliations:** 1 A2M, Amsterdam, The Netherlands; 2 Ecole de Santé Publique, Université Libre de Bruxelles, Bruxelles, Belgium

**Keywords:** Drug price, Cost-effectiveness, Budget impact, Economic valuation

## Abstract

**Background:**

Innovative orphan drugs often have an incremental cost-effectiveness ratio (ICER) which is higher than the maximum threshold for reimbursement. Payers have limited budgets and often cannot pay the full price of a new product, but pharmaceutical and biotechnology companies require a minimum price to satisfy their investors. The objective of this study was to present a possible solution to bridge this pricing gap by having early phase price agreements, which reduce the risk for investors.

**Methods:**

We used a Pricing Model, which determines the minimum (break-even) price of an innovative drug from an investor’s perspective. This model is based on economic valuation theory, which uses the expected free cash flows and the required cost of capital. We selected two orphan drugs with a positive clinical assessment and an ICER higsher than the Dutch maximum threshold of €80,000 per QALY gained to use as examples in the model: Spinraza for spinal muscular atrophy and Orkambi for cystic fibrosis.

RESULTS: The results show that early pricing agreements before phase III trials can substantially lower the drug price resulting from a lower cost of capital. The minimum price for orphan drugs can be reduced by 27.4%, when cost of capital decreases from 12 to 9%. An additional adjustment of other critical parameters due to early pricing agreements (lower probabilities of phase III failure and lower research and development (R&D) costs) can further reduce the minimal price by 62.8%.

**Conclusion:**

This study shows that earlier timing of price negotiations resulting in an agreement on drug price can substantially lower the minimal price of orphan drugs for the investor.

## Background

Nationwide coverage systems for new medicines informed by centralized decision making on their efficacy, and in some cases value for money, were appropriate for medicines launched in the 1970s and 1980s. These were often relatively low value products such as antibiotics and antidepressants. However, in the 1990s new classes of medicinal products entered the market, for example biologicals with completely different mechanisms of action, higher production costs, and much higher prices. As a result, many new innovative medicinal products increasingly have an incremental cost-effectiveness ratio (ICERs), which is higher than the maximum threshold for reimbursement. In the Netherlands, the upper threshold is €80,000 per quality-adjusted life year (QALY), which is the maximum amount that the Dutch society is willing to pay for a gain in one QALY [1]. Once the drug price exceeds the national threshold, authorities in many European Union (EU) markets initiate price negotiations before making a final reimbursement decision. The ICER often guides this price negotiation leading to a required discount of the drug price. This discount applies to the drug price, which should lower the ICER to meet the threshold. However, these discounts may result in prices of medicines that are unacceptably low for pharmaceutical companies and investors, which may have major immediate financial consequences for the pharmaceutical industry [2]. For example, in 2017 the Dutch Ministry of Health required a price discount of 80% for Orkambi (lumacaftor/ivacaftor combination therapy), a new medication for cystic fibrosis (CF), in order to reduce the ICER from €170,000 to the maximum threshold of €80,000 [3]. The resulting €34,000 corresponds with such a huge price discount that investors in medical innovation may withdraw and will switch to other markets yielding a higher return on investment. Therefore, the current pricing policies may have major long-term detrimental consequences for patient and healthcare providers.

Because health authorities consider medical innovation as the responsibility of entrepreneurs in the pharmaceutical and biotechnology markets, they cannot ignore the demand for a minimum return on investment for investors that drives this market. The size of the market for orphan drugs is usually quite small because of the limited number of potential patients. However, the total costs for the development of orphan drugs may be comparable to the R&D costs for the development program of non-orphan drugs, at circa US$700 million [4]. Consequently, the prices for orphan drugs ought to be substantially higher than for the other drugs, because the costs for development can only be regained on a much smaller patient population. Recently, we introduced a Pricing Model to determine the price of new and innovative drugs from the investor’s perspective, which is based on economic valuation theory. A follow-up study showed that this Pricing Model is a useful tool for health authorities to assess the price of orphan drugs with an ICER higher than the threshold. The model showed that nearly 80% of orphan drug prices in this analysis were reasonable from the investor’s perspective [5].

Finally, orphan drugs with an acceptable ICER can still have significant impacts on the national drug budgets, as an ICER below the threshold is not equivalent to a measure of affordability. Although society is willing to pay for these drugs based on an acceptable ICER, the payer may not be able to afford these drugs due to constrained budgets [6]. We must accept this current deadlock and understand the constraints from the perspective of both payers and pharmaceutical companies: payers have limited budgets and cannot afford the full price, but pharmaceutical companies require a minimum price to satisfy their investors.

Therefore, the objective of this paper is to present a possible solution to bridge the current gap between the required price for orphan drugs from the investor’s perspective and the acceptable drug price from a payer and societal perspective. The primary hypothesis is that early phase price agreements will reduce the risk for investors, translating into a lower minimum drug price. In addition, we will explore the potential positive impact of pricing agreements on other critical parameters, for example lower probabilities of phase III failure and lower research and development (R&D) costs.

## Methods

### Concept

The concept of the new Pricing Model is briefly described here, but full details are provided in two previous publications [7, 8]. The present value equation is based on the discounted cash flows and the cost of capital [7, 8].

1 $$ \mathrm{NPV}={\mathrm{CF}}_1/\left({\left(1+\mathrm{r}\right)}^1+{\mathrm{CF}}^2/\right({\left(1+\mathrm{r}\right)}^2+---+{\mathrm{CF}}_{\mathrm{n}}/\Big({\left(1+\mathrm{r}\right)}^{\mathrm{n}} $$

Where

NPV = net present value

CF = (free) cash flow

n = the time in years before the future cash flow occurs

r = cost of capital

Cash flows from operations correspond with drug sales, and expenditures for R&D, production and marketing. The cost of capital is the minimum rate that an investor expects to earn when investing in a project, which corresponds with the cost of capital in the pharmaceutical market [9]. The discounted cash flow method allows the calculation of the break-even (BE) price for the new innovative drug which leads to a net present value of zero. Consequently, this BE price reflects the minimum drug price from the investor’s perspective. For example, the price of €34,000 for Orkambi which was requested by the Dutch health authorities, leads to a negative net present value and therefore would not be acceptable for an investor. On the other hand, if the BE price is much lower than the actual price, economic valuation theory would not consider the actual drug price fair.

The input parameters of the Pricing Model are listed in Table 1. We assumed that the product was registered at year eight after patent registration and reimbursement was obtained within 1 year, leaving 11 years for actual sales before the patent expired. The allocation of R&D failures to successful drugs obtaining European Medicines Agency (EMA) or Food and Drug Administration (FDA) approval is an important element in the valuation according to the principles of economic valuation. The probabilities of failure during the development phases (phase I, II and III) are derived from published literature [4, 10].

**Table 1:** The input parameters for current and early timing of price negotiations

Model parameter			Remarks
Cost of development (US$ million)	US$701 million	€660 million	Not specific for orphan disease or orphan drugs in oncology
Cost pre-clinical	US$217 million	€205 million	
Phase I	US$84 million	€79 million	
Phase II	US$142 million	€134 million	
Phase III	US$190 million	€168 million	
Phase IV	US$68 million	€64 million	
Years of development & approval	8 years		
Net patent life	12 years		
Population	Western markets: 947.1 million		
	Larger global market: 1743.4 million		
Period between registration and reimbursement	1.5 years		Based on increasing hurdles, especially for high priced orphan drugs (reimbursement) + pricing negotiations
Net patent life	12 years		
Uptake	80% from first year		Uptake is higher in orphan diseases than other drugs
Cost of revenue	40%		
Probability success			
Phase I to II	70%		
Phase II to III	39%		
Phase III to approval (FDA/EMA)	69%		
Reimbursement	90%		Based on increasing hurdles, especially for high priced orphan drugs (reimbursement) + pricing negotiations
Cost of capital	12%		

### Early phase price negotiations

Sensitivity and scenario analyses in a previous study showed that the cost of capital, market size, and risk of failure of clinical trials were critical parameters in the Pricing Model [5]. Delay due to the reimbursement procedure and probability of reimbursement failure may also have a substantial impact on the results. These findings resulted in the development of another policy model for reimbursement and pricing of drugs in this study. The focus of this model is on the reduction of risk for the investor, which lowers the cost of capital. Based on the current time horizon, the risk for the investor is high because the decision on reimbursement and the final price of the drug is made 8 years after the initial investment. Therefore, we hypothesise that the risk for the investor would be substantially lower if the price negotiations were conducted earlier, and therefore the cost of capital would also be lower. This would result in a lower BE price (i.e. minimum price) at no cost to any of the stakeholders (investor, payer or society). The Pricing Model does not exclude the subsequent uncertainty in the possible events after the early phase price agreement, for example failure of phase III clinical trials or failure of reimbursement because of insufficient clinical benefit or an ICER higher than the threshold. But even if these probabilities remain similar, an early phase agreement reduces the risk for the investor.

The current price negotiations occur after health technology assessment (HTA) bodies provide their assessment report of the reimbursement dossier to their national health authorities. If the HTA bodies are convinced of the clinical benefit but the ICER exceeds the threshold, health authorities are likely to initiate price negotiations. The required discounted drug price is often based on the ICER threshold, which is usually much lower than the actual price and also often the BE price for the investor based on the Pricing Model, as we showed for Orkambi.

The timing of current price negotiations could also be questioned from an ethical perspective. Once the drug has been registered and the health authorities agree there is a clinical benefit the patient would soon expect access to the new drug. Although the outcomes of price negotiations may be justified from an economic perspective, this may not be understood or accepted in the public domain, especially by patients and their relatives in this final step before patient access. The anticipation of these reactions in the public domain may also include a subjective and emotional component in the price negotiation process. This may disturb an optimal price negotiation process and weakens the bargaining power of the health authorities. Hence the current timing of price negotiations may lead to inadequate pricing decisions from a societal perspective.

We therefore considered price negotiations resulting in an agreement on drug price before the initiation of phase III trials based on the outcomes of phase II trials. Given that these pricing discussions are 3 to 4 years before the actual launch, the impact of a negative decision on public perception will be much smaller and will raise fewer ethical concerns.

Table 1 shows the definition of the parameters for the current and early timing of price negotiations. Harrington provides estimates of the cost of capital, which are 9% for pharmaceuticals, and 12% for biotechnology [9]. In the example below, a 12% cost of capital was used in all phases for the current and late pricing negotiations, because biotechnology companies are responsible for development of Spinraza and Orkambi. However, the outcomes of early price negotiations reduce the subsequent risk for the investor substantially after phase II. We assume that the risk decreases from 12 to 9%, 7 and 5%, reflecting values between 12% and a risk-free interest of 4% (https://www.multpl.com/10-year-treasury-rate/table/by-year). This 4% is the average risk-free interest based on the period from 2007 to 2010, which is the T = 0 moment for investors in the early phase of development of Spinraza and Orkambi. The focus of the base case analysis is only on lowering the cost of capital, but in additional scenario analyses we explored the impact of early price negotiations on other critical parameters, e.g. lower probabilities of phase III failure and lower R&D costs. The potential number of patients is based on the global potential number of patients.

Finally, the model includes eligibility and uptake. Patients may not be eligible for treatment because of contra-indications or a risk of adverse events. In addition, not all eligible patients are treated as soon as the drug is available. In the financial analysis, this uptake or diffusion generally increases from time of launch over the follow-up period [11].

### Application

We selected only first-in-class innovative orphans with a positive clinical assessment and an ICER exceeding €80,000 per QALY gained as examples in the model. These were Spinraza for spinal muscular atrophy and Orkambi for cystic fibrosis (Table 1) [12]. We applied the discounted cash flow method to the projections of these expensive orphan drugs in The Netherlands in a previous study based on information from the Dutch National Health Care Institute (“Zorginstituut Nederland” - Zin) (Table 1) [5] (https://www.zorginstituutnederland.nl/). In this previous analysis, the actual prices of Orkambi and Spinraza were much higher than the calculated BE prices based on our Pricing Model: Spinraza was actually priced at €240,000 with a BE price of €95,860, and Orkambi was actually priced at €169,386 with a BE price of €65,861. These findings indicated that further exploration was required to judge if the actual prices really were necessary to satisfy investors, for example production costs may be substantially higher for these drugs. The objective of this study was not to challenge the actual prices for Spinraza and Orkambi, but to present a solution to the current gap between the required and affordable price for orphan drugs from the perspective of the investor and the payer. To assess this gap, the R&D costs were calibrated such that the actual prices equalled the break-even prices for both drugs by applying the same adjustment factor to all phases of development for each drug separately.

## Results

### Base case analysis

Table 2 shows the results of the sensitivity and scenario analyses for Spinraza and Orkambi for the current late price negotiations. The cost of capital, market size, and risk of failure of clinical trials are the most critical parameters in the model. For example, a decrease from 12 to 9% in cost of capital would mean the price of Spinraza could be lowered by €54,739 and €46,034 for Okambi. Conversely, when the cost of capital was 18%, the price of Spinraza would have to increase by €223,042 and €138,9919 for Okambi to reach the BE price.

**Table 2:** The results of the sensitivity and scenario analyses (late price negotiations)

Parameter	Scenario	Price			
		Spinraza		Orkambi	
Actual price (BE price)		€240,000		€169,386	
**Scenario**		BE price	change	BE price	change
Global market	large market	€175,768	-€ 64,232	€117,030	-€52,356
Incidence (per 100,000)	min. - 20%	€318,000	€ 78,000	€211,733	€42,347
	min. − + 20%	€212,000	-€ 28,000	€141,155	-€28,231
Off-label use	min. 2.5% (assumption)	€248,195	€ 8195	€167,837	-€1549
	max. 5.9% (assumption)	€242,286	€ 2286	€170,963	€1577
Growth population	min. 0% (assumption)	€290,743	€ 50,743	€176,719	€7333
	max. 2.5% (assumption)	€226,133	-€ 13,867	€163,301	-€6085
Cost of capital	Pharma −9%	€185,261	-€ 54,739	€123,352	-€46,034
	Assumption 18%	€463,042	€ 223,042	€308,305	€138,919
Probability success phase I-II	min. - 20%	€301,970	€ 61,970	€201,060	€31,674
	min. − + 20%	€222,686	-€ 17,314	€148,270	-€21,116
Probability success phase II -III3	min. - 20%	€313,044	€ 73,044	€208,433	€39,047
	min. − + 20%	€215,304	-€ 24,696	€143,355	-€26,031
Probability success phase III - registration	min. - 20%	€317,383	€ 77,383	€211,322	€41,936
	min. − + 20%	€212,411	-€ 27,589	€141,429	-€27,957
Probability reimbursement	min. (assumption) - 80%	€321,975	€ 81,975	€214,379	€44,993
	max. (assumption) -100%	€206,064	-€ 33,936	€190,559	€21,173
Eligible	min. (assumption) - 80%	€286,200	€ 46,200	€190,559	€21,173
	max. (assumption) -100%	€228,960	-€ 11,040	€152,447	-€16,939
Cost marketing	min. - 20%	€218,057	-€ 21,943	€145,188	-€24,198
	min. − + 20%	€305,280	€ 65,280	€203,263	€33,877
Costs R&D - preclinical	min. - 20%	€225,700	-€ 14,300	€150,277	-€19,109
	min. − + 20%	€283,100	€ 43,100	€188,495	€19,109
Costs R&D - phase I	min. - 20%	€245,044	€ 5044	€163,157	-€6229
	min. − + 20%	€263,756	€ 23,756	€175,615	€6229
Costs R&D - phase II	min. - 20%	€245,541	€ 5541	€163,487	-€5899
	min. − + 20%	€263,259	€ 23,259	€175,285	€5899
Costs R&D - phase III	min. - 20%	€250,929	€ 10,929	€167,075	-€2311
	min. − + 20%	€257,871	€ 17,871	€171,697	€2311
Costs R&D - phase IV	min. - 20%	€253,906	€ 13,906	€169,057	-€329
	min. − + 20%	€254,894	€ 14,894	€169,715	€329
Uptake	min. (assumption) - 90%	€290,743	€ 50,743	€193,584	€24,198
	max. (assumption) - 95%	€226,133	-€ 13,867	€150,565	-€18,821
Proportion in clinical trials	min. (assumption) - 5%	€267,789	€ 27,789	€178,301	€8915
	max. (assumption) - 10%	€279,560	€ 39,560	€188,207	€18,821

Table 3 shows the results of the base case analysis, which only assesses the impact of early timing of price negotiations on a lower cost of capital. The cost of capital for the base case analysis is 12% for late price negotiations, whereas we applied 9, 7 and 5% for the assessment of early price negotiations. The results reflect the difference in the BE price for Spinraza and Orkambi between late (current situation) and early price negotiations before phase III of the clinical development program.

**Table 3:** The results of the base case analyses comparing timing of price negotiations

		Spinraza			Orkambi		
Actual price		€ 240.000			€ 169,386		
Cost of capital		9%	7%	5%	9%	7%	5%
> Phase III	BE price	€ 203,449	€ 181,040	€ 160,202	€ 143,589	€ 127,774	€ 113,066
	Change	−15.2%	−24.6%	−33.2%	−15.2%	− 24.6%	− 33.2%
>Phase II	BE price	€ 188,394	€ 159,123	€ 133,553	€ 132,964	€ 112,305	€ 94,259
	Change	− 21.5%	−33.7%	−44.4%	−21.5%	− 33.7%	−44.4%

The results show that early pricing negotiations after phase II can reduce the BE price for Spinraza by 21.5% from €240,000 to €188,394 for a 9% cost of capital. This is a conservative scenario as the 9% corresponds with cost of capital for pharmaceutical companies. To show the full potential impact of early pricing negotiations and sensitivity to cost of capital, we also performed analyses for 7 and 5% cost of capital. This reduced the BE price by 33.7 and 44.4% respectively. The same percentage reduction in the BE price was found for Orkambi.. For both drugs, later price negotiations after Phase III reduced the BE price by 15.2%, which is still substantial, but much lower than the 21.5% after Phase II.

### Scenario analyses

Table 4 shows the results of additional scenario analyses on the price of Spinraza for other critical parameters. The percentage change shown for Spinraza was the same for Okambi as we show in Table 3. We have estimated how the price would change when the period between registration and reimbursement reduces from 1.5 to 0.5 years, and the uptake increases from 80 to 90% and 95%. The other scenario analyses are based on 5 and 10% improvements on the other parameters (Table 4). We also added scenario analyses combining a minimum (e.g. 5%) and maximum (e.g. 10%) improvement on all parameters. The break-even price decreased by 62.8% from €240,000 to €89,336 when the cost of capital of was 9% (in the maximum scenario). The 7 and 5% cost of capital lead to a BE price of €76,109 and €64,422 respectively in this maximum scenario, which corresponds with a 68.3 and 73.2% price decrease.

**Table 4:** The results of the scenario analyses comparing timing of price negotiations - Spinraza

Actual price		€ 240.000					
		absolute			change		
**Costs**		9%	7%	5%	9%	7%	5%
Phase III	5%	€ 187,721	€ 158,536	€ 133,044	−21.8%	−33.9%	−44.6%
	10%	€ 187,047	€ 157,949	€ 132,535	−22.1%	−34.2%	−44.8%
Phase IV	5%	€ 188,285	€ 159,019	€ 133,454	−21.5%	−33.7%	−44.4%
	10%	€ 188,176	€ 158,914	€ 133,355	−21.6%	−33.8%	−44.4%
Total	5%	€ 187,611	€ 158,431	€ 132,945	−21.8%	−34.0%	−44.6%
	10%	€ 186,829	€ 157,740	€ 132,337	−22.2%	−34.3%	−44.9%
**Probabilities**							
Phase III to IV	5%	€ 179,527	€ 151,645	€ 127,288	−25.2%	−36.8%	−47.0%
	10%	€ 171,466	€ 144,847	€ 121,593	−28.6%	−39.6%	−49.3%
Reimbursement	5%	€ 170,879	€ 144,329	€ 121,137	−28.8%	−39.9%	−49.5%
	10%	€ 155,698	€ 131,507	€ 110,375	−35.1%	−45.2%	−54.0%
Eligibility	5%	€ 179,423	€ 151,546	€ 127,194	−25.2%	−36.9%	−47.0%
	10%	€ 171,267	€ 144,657	€ 121,412	−28.6%	−39.7%	−49.4%
All	5%	€ 155,082	€ 130,997	€ 109,956	−35.4%	−45.4%	−54.2%
	10%	€ 128,825	€ 108,826	€ 91,354	−46.3%	−54.7%	−61.9%
**Cost marketing**							
	5%	€ 182,317	€ 153,990	€ 129,245	−24.0%	−35.8%	− 46.1%
	10%	€ 176,619	€ 149,178	€ 125,206	−26.4%	−37.8%	−47.8%
**Time saving (months)**	6	€ 176,990	€ 150,194	€ 126,641	−26.3%	− 37.4%	−47.2%
	12	€ 166,888	€ 142,214	€ 120,409	− 30.5%	−40.7%	−49.8%
**Uptake (base 80%)**	90%	€ 158,648	€ 133,998	€ 112,466	−28,8%- 33.9%	−39.9%- 44.2%	−49.5%- 53.1%
	95%	€ 167,461	€ 141,443	€ 118,714	−35,1%- 30.2%	−45.2%- 41.1%	−54.0%- 50.5%
**Combined**							
Minimum		€ 124,805	€ 105,897	€ 89,279	−48.0%	−55.9%	−62.8%
Maximum		€ 89,336	€ 76,109	€ 64,422	−62.8%	−68.3%	−73.2%

## Discussion

The high impact of increasing new orphan drugs on the healthcare budgets, may endanger the affordability of these important drugs for many patients. As more orphan drugs are being developed, healthcare budgets are increasingly unable to cover the costs of these expensive treatments. On the other hand, the prices for most orphan drugs seem justified from the investor’s perspective based on the expected free cash flows and the required cost of capital [5]. This study presents a proposal to address the growing issue of the costs of biopharmaceuticals for orphan diseases. The Pricing Model shows that entering into early price agreement possible following results of Phase II trials could lead to a reduction in capital costs and subsequently company/investor price expectations at time of marketing. The main advantage is that health authorities pay at least a 25–30% lower drug price. The company obtains a lower drug price, but there is also lower payment to the investor, so there may be actual no loss for the company. For the investor, the return on investment is lower, but on the other hand there is also less investor’s risk, so the lower drug price is also not a real loss for the investor.

In this study, we have shown that the cost of capital is a key driver of drug price, which reflects the risk for the investor. In our 20-year time-horizon example, this risk is high because the decision on reimbursement and the actual price occurs 8 years after the initial investment. Therefore the objective of this study was to assess whether early price negotiations before phase III, could lower the minimal price to satisfy the investor. We applied the approach to Spinraza and Orkambi, which were heavily scrutinized by the health authorities because of their high budget impact and ICERs exceeding the threshold. The results show that early pricing negotiations before phase III can substantially lower the minimal price for the investigated orphan drugs when we consider a lower cost of capital. The minimal price for orphan drugs can be reduced by 21.5% when the cost of capital is 9%. A cost of capital of 7 and 5% reduces the minimal price by 33.7 and 44.4% respectively. However, the 9% cost of capital (reflecting the cost of capital for pharmaceuticals), may be too conservative and the 5%, which is close to the 4% risk-free rate, is too optimistic. Hence 7% may be most reasonable cost of capital for interpretation of these initial results. The assessment of the most appropriate cost of capital requires further research, but the current analyses show many potential mechanisms for lowering drug prices, regardless of the rate of cost of capital. For example, the timing of price negotiations is a sensitive parameter and the model results show that negotiations before Phase III have a more favourable impact on lowering the minimum price than later negotiations.

As we mentioned previously, in 2017 the Dutch Ministry of Health requested a discount of 80% for Orkambi. Our analysis shows that the minimum price for orphan drugs can be reduced by 27.4%, and impact on other critical parameters can further reduce the minimal price by 62.8%. Early price negotiations would have reduced the gap from 80 to 17.2% facilitating a mutual agreement. In the end, Orkambi has been reimbursed, which means that Dutch health authorities agreed on a drug price, which is secret, but certainly is higher than the requested 80% discount, and consequently the ICER for Orkambi is probably higher than the threshold. Hence early phase price negotiations for Orkambi would have resulted in a lower price with an ICER probably closer to the threshold.

The choice of early price negotiations before phase III corresponds with the timing of conditional marketing authorisation (CMA) by the Committee for Orphan Medicinal Products (COMP), which is the EMA’s committee responsible for recommending orphan designated medicines for rare diseases. **An** assessment of conditional marketing authorisations by the EMA over 10 years, highlighted that approximately half of all the clinical studies for conditional authorization were phase II [13]. This report also recommended improved early dialogue with relevant stakeholders, in particular Health Technology Assessment Bodies, which means that there is organizational structure for including our proposed early pricing negotiations.

Aside from investor risk, there are other parameters that may have a positive impact on the BE price. An early mutual agreement between the biotech company and health authorities on the drug price before phase III may lead to an additional commitment by the health authorities for the next trial phases until registration and after launch. This may contribute to improvements in clinical design and an increase in the logistical efficiencies of the clinical trials, and may lead to lower probabilities of phase III failure and lower R&D costs. In addition, we may expect a higher probability of reimbursement, higher eligibility and uptake, and a reduction in time between registration and reimbursement. The impact of early price negotiations on these other parameters can also reduce the minimum price, but the impact is less substantial. However, as shown in the scenario analyses, when all parameters are changed simultaneously, the minimum price further decreases from 21.5 to 62.8% for a cost of capital of 9%. The investor may include this positive impact on other critical parameters in his assessment, and may be willing to accept even a lower cost of capital because of this additional lower risk, which was not included in this analysis.

The same report by the EMA (https://www.zorginstituutnederland.nl/) provides support for the impact of these other parameters, as it recommends that post-authorisation activities should be planned carefully and timely, facilitating rapid completion of additional studies and availability of comprehensive data.

The Pricing Model provides a global minimal price for a drug. The results from this model have shown that the impact of early phase price negotiations cannot be based on separate negotiations with pharma companies by each country, because each separate country-specific adjustment of the listed input parameters does not have any impact on the global drug price. Early phase price negotiations only have practical value if price negotiations take place at least at an EU level. As budget constraints are an issue in most large markets, a semiglobal process would be preferred.

The concept of Pricing Model itself may be valuable tool for assessing if a drug price is reasonable, but that actual data, e.g., R&D costs and actual clinical benefit, certainly needs further research. However, the Pricing Model in this study is not used to judge the price of Spinraza or Orkambi, but it is used to reflect the invisible hand in the international financial markets in a free market economy, regardless of the use of the Pricing Model. This analysis shows that the drug price is extremely sensitive to early price negotiations and therefore any deviations from the actual R&D costs or probabilities of failure of clinical trial would not change the strong trend from our analysis and our conclusions.

## Conclusion

This study shows that earlier timing of price negotiations resulting in an agreement on drug price can reduce the risk for the investor, which can substantially lower the minimum price for the drug. The Pricing Model shows the potential of price reduction is 21.5% for a conservative cost of capital of 9%, but can extend to 62.8% in the most optimistic scenario.

## Data Availability

not applicable

## Abbreviations

BE: Price
CF: Cystic fibrosis
CMA: Conditional marketing authorisation
COMP: Committee for Orphan Medicinal Products
EMA: European Medicines Agency
EU: European Union
FDA: Food and Drug Administration
ICER: Incremental cost-effectiveness ratio
HRA: Health technology assessment
NPV: Net present value
R&D: Research and development
Zn: Zorginstituut Nederland

## References

[1] Reckers V, van Exel NJA, Brouwer WBF (2018). Looking back and moving forward: on the application of proportional shortfall in healthcare priority setting in the Netherlands. Health Policy.

[2] Nuijten M, Vis J (2018). Economic comments on proposal for a novel cancer drug pricing model. Nat Rev Clin Oncol.

[3] Herbeoordeling lumacaftor/ivacaftor (Orkambi). 2016. Reference 2016136882.Zorginstituut Nederland. https://www.zorginstituutnederland.nl/publicaties/adviezen/2016/12/15/gvs-advies-lumacaftor-ivacaftor-orkambi-bij-cystische-fibrose-cf-bij-patienten-van-12-jaar-en-ouder-die-homozygoot-zijn-voor-de-f508del-mutatie-in-het-cftr-gen-herbeoordeling.

[4] Sloand J (2014). Assessing the financial viability of stratified medicine using decision tree analysis. Working paper.

[5] Nuijten M, Fugel HJ, Vis J (2018). Evaluation and valuation of the price of expensive medicinal products: application of the discounted cash flow to orphan drugs. J Rare Dis Disord.

[6] Uyl-de Groot CA, Löwenberg B (2018). Sustainability and affordability of cancer drugs: a novel pricing model. Nat Rev Clin Oncol.

[7] Nuijten MJC, Vis J (2016). Evaluation and valuation of innovative medicinal products. J Rare Dis Res Treat.

[8] Nuijten M, Capri S (2020). Pricing of orphan drugs in oncology and rare diseases. J Mark Access Health Policy.

[9] Harrington S (2009). Cost of Capital for pharmaceutical, biotechnology, and medical device firms.

[10] Fugel HJ, Nuijten M, Postma M (2016). Economic viability of stratified medicine concepts: an investor perspective on drivers and conditions that favour using stratified medicine approaches in a cost-contained healthcare environment. New Biotechnol.

[11] Nuijten MJ, Mittendorf T, Persson U. Practical issues in handling data input and uncertainty in a budget impact analysis. Eur J Health Econ. 2010. 10.1007/s10198-010-0236-4. 10.1007/s10198-010-0236-4 PMC3078307 20364289

[12] Bijlage 1 Horizonscan VWS - uittreksel. 2016. https://www.horizonscangeneesmiddelen.nl/binaries/content/assets/horizonscan/horizonscan-vws-uittreksel-december-2016.pdf.

[13] Conditional marketing authorisation. Report on ten years of experience at the European Medicines Agency. EMA/471951/2016 © European Medicines Agency. 2017. https://www.ema.europa.eu/en/documents/report/conditional-marketing-authorisation-report-ten-years-experience-european-medicines_agency_en.pdf.

